# A High-Quality CdSe/CdS/ZnS Quantum-Dot-Based FRET Aptasensor for the Simultaneous Detection of Two Different Alzheimer’s Disease Core Biomarkers

**DOI:** 10.3390/nano12224031

**Published:** 2022-11-16

**Authors:** Xingchang Lu, Xiaoqi Hou, Hailin Tang, Xinyao Yi, Jianxiu Wang

**Affiliations:** 1 Hunan Provincial Key Laboratory of Micro & Nano Materials Interface Science, College of Chemistry and Chemical Engineering, Central South University, Changsha 410083, China; 2 School of Chemistry and Material Science, Hangzhou Institute for Advanced Study, University of Chinese Academy of Sciences, 1 Sub-lane Xiangshan, Hangzhou 310024, China; 3 Key Laboratory of Intelligent Sensing Materials and Chip Integration Technology of Zhejiang Province, Hangzhou Innovation Institute, Beihang University, Hangzhou 310052, China; 4 SunYat-sen University Cancer Center, State Key Laboratory of Oncology in South China, Collaborative Innovation Center for Cancer Medicine, Guangzhou 510060, China

**Keywords:** quantum dots, Aβ oligomers, tau protein, simultaneous detection, aptasensor

## Abstract

The simultaneous detection of two different biomarkers for the point-of-care diagnosis of major diseases, such as Alzheimer’s disease (AD), is greatly challenging. Due to the outstanding photoluminescence (PL) properties of quantum dots (QDs), a high-quality CdSe/CdS/ZnS QD-based fluorescence resonance energy transfer (FRET) aptasensor for simultaneously monitoring the amyloid-β oligomers (AβO) and tau protein was proposed. By engineering the interior inorganic structure and inorganic–organic interface, water-soluble dual-color CdSe/CdS/ZnS QDs with a near-unity PL quantum yield (>90%) and mono-exponential PL decay dynamics were generated. The π–π stacking and hydrogen bond interaction between the aptamer-functionalized dual-color QDs and gold nanorods@polydopamine (Au NRs@PDA) nanoparticles resulted in significant fluorescence quenching of the QDs through FRET. Upon the incorporation of the AβO and tau protein, the fluorescence recovery of the QDs-DNA/Au NRs@PDA assembly was attained, providing the possibility of simultaneously assaying the two types of AD core biomarkers. The lower detection limits of 50 pM for AβO and 20 pM for the tau protein could be ascribed to the distinguishable and robust fluorescence of QDs and broad spectral absorption of Au NRs@PDA. The sensing strategy serves as a viable platform for the simultaneously monitoring of the core biomarkers for AD and other major diseases.

## 1. Introduction

Alzheimer’s disease (AD) is a progressive and severe neurodegenerative disorder of the brain characterized by memory loss, cognitive dysfunction, behavioral disability, and irreversible brain damage, which drastically affects public health [1]. The pathological cascade of AD lasts for at least 10–15 years prior to the onset of clinical symptoms [2]. It has been confirmed that amyloid plaques and neurofibrillary tangles, which are formed largely by fibrillar forms of amyloid-β (Aβ) peptide and hyperphosphorylated tau protein, may contribute to the AD pathogenesis [3,4]. Among the various aggregated forms, the soluble Aβ oligomer (AβO), rather than the small Aβ monomer (AβM) and insoluble Aβ fibril (AβF), is the most neurotoxic form in vivo [5]. Thus, it is extremely desirable to monitor the core biomarkers of the AβO and tau protein for the point-of-care diagnosis and study of the pathogenesis of AD.

In recent years, numerous efforts have been made to develop reliable methods for the quantitative and accurate determination of AD biomarkers, including but not limited to electrochemistry [6,7], electrochemical luminescence [8,9], photo-electrochemistry [10,11], colorimetry [12,13], surface-enhanced Raman scattering [14], surface plasmon resonance [15], and so on. Recently, QD-based FRET and electrochemiluminescence resonance energy transfer (ECL-RET) systems have been constructed. For example, Jia’s group reported a dual-wavelength ratiometric ECL-RET aptasensor for the detection of the Aβ protein [9], which exhibited a wider concentration range and superior sensitivity and selectivity for Aβ analysis. Most of the above methods aimed to assay one kind of AD biomarker, with either a low sensitivity or requirements for sophisticated instrumentation. It is known that almost all the major diseases are accompanied by the abnormal regulation of multiple biomarkers, and the detection of a single biomarker may cause a false diagnosis [16,17]. As an alternative, the fluorescence resonance energy transfer (FRET)-based methods, in which the energy from the excited fluorophore (energy donor) is transferred to the quencher (energy acceptor) in close proximity (within 10 nm), possesses a wide applicability in biological assays [18,19,20,21,22,23].

Colloidal quantum dots (QDs) serve as an unprecedented class of photo-emissive materials for optical biosensing due to their unique characteristics, such as their size-dependent fluorescent emission, broad excitation and narrow emission spectra, high emission brightness, and excellent optical and thermal stability [24,25,26,27]. Furthermore, the specific photophysical properties of QDs, such as their fluorescence blinking, quantum yield, temperature-dependent characteristics, etc., can affect their optical and optoelectronic applications [28,29,30,31,32]. Recently, tremendous advances in the fabrication of CdSe/CdS core/shell QDs with nearly ideal optical properties have been achieved [33,34]. However, these QDs were all synthesized in nonaqueous media and are not suitable for biosensing applications [35]. A variety of strategies have been proposed to construct water-soluble QDs, such as their encapsulation in micelles [36], coating with silica [37], and ligand exchange [38]. The first two strategies led to the formation of QDs of large hydrodynamic sizes, and the increased overall distance between the donor–acceptor pairs lowered the FRET efficiency, which limited the FRET-based biosensing applications [39]. By contrast, ligand exchange is a relatively simple procedure, and the compact QDs formed were better suited to FRET-based applications. It is worth noting that a large variety of surface traps usually existed on the water-soluble QDs [40,41]. To isolate these traps from the photogenerated excitons in the QDs, it was necessary to epitaxially grow an additional layer of inorganic shell with a large bandgap, such as a ZnS shell [42,43,44]. However, due to the large lattice mismatch (12%) between the CdSe core and ZnS shell, the intermediate layer of CdS was introduced to relieve the lattice strain [45]. Thus, the CdSe/CdS/ZnS core/shell/shell QDs acted as an ideal candidate for biosensing applications.

Gold nanorods (Au NRs) have been widely used in biological applications due to their high extinction coefficient and shape and size controllability [46,47]. It was found that the surface of Au NRs can be derived from various species, such as dopamine, and the resultant polydopamine-capped gold nanorods (Au NRs@PDA) were functionalized with amino, hydroxyl, and quinone groups [48]. These functional groups on the Au NRs@PDA then served as a bridge for the combination with single-stranded DNA through hydrogen bonding and π–π stacking [49,50,51]. Furthermore, the broad spectral absorption of the Au NRs@PDA provided the possibility of designing fluorescence-quenched assays.

**Scheme 1:**
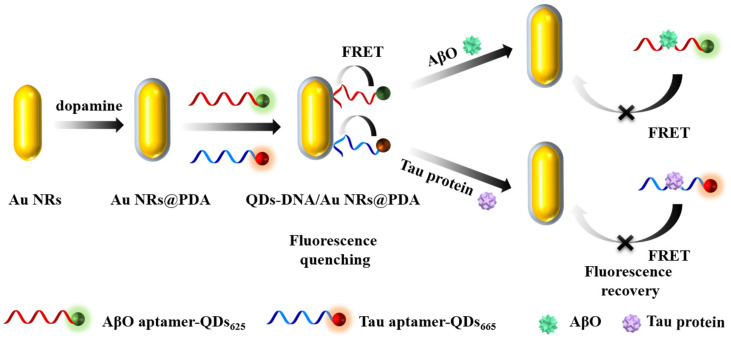
Schematic illustration of the simultaneous detection of AβO and tau protein using the QDs-based FRET aptasensor.

Using dual-color QDs as energy donors and Au NRs@PDA as energy acceptors, a simple and sensitive FRET aptasensor for the simultaneous detection of the AβO and tau protein was proposed (Scheme 1). Two color-emitting CdSe/CdS/ZnS core/shell/shell QDs with ideal optical properties were synthesized and functionalized with aptamers to the AβO and tau protein, respectively. Through π–π stacking and hydrogen bond interaction, the QDs-DNA/Au NRs@PDA assembly was constructed, which triggered the FRET between the QDs-DNA and Au NRs@PDA due to their close proximity. However, the specific binding of the aptamers to the AβO and tau protein led to the detachment of the QDs-DNA from the surface of Au NRs@PDA. Thus, the quenched fluorescence signals were remarkably recovered. By monitoring the change in the fluorescence signals of the QDs-DNA under single-wavelength excitation, the simultaneous detection of two different AD core biomarkers was achieved.

## 2. Materials and Methods

### 2.1. Chemicals and Reagents

Silver nitrate (AgNO_3_), SH-PEG-CH_3_ (methoxy poly(ethylene glycol) thiol, MW = 5000), bovine serum albumin (BSA), selenium powder (Se, 200 mesh, 99.999%), cadmium oxide (CdO, 99.998%), and sulfur powder (S, 99.98%) were obtained from Sigma-Aldrich (St. Louis, MO, USA). Mercaptopropionic acid (MPA, >99%), tetramethylammonium hydroxide (TMAH, 25% *w/w* in methanol), myristic acid (98%), stearic acid (>90%), and 1-octadecene (ODE, 90%) were purchased from Alfa Aesar (Shanghai, China). 1,1,1,3,3,3-Hexafluoroisopropanol (HFIP) was acquired from Shanghai Macklin Biochemical Co., Ltd. (Shanghai, China). Hydrogen tetrachloroaurate trihydrate (HAuCl_4_·3H_2_O), sodium borohydride (NaBH_4_), cetyltrimethylammonium bromide (CTAB), dimethyl sulfoxide (DMSO), ascorbic acid, and other organic solvents were obtained from Sinopharm Chemical Reagent Co., Ltd. (Shanghai, China). All the chemicals were used directly, without any purification. Aβ (1–40) peptide (catalog number: HY-P0265) was purchased from MedChemExpress (Monmouth Junction, NJ, USA). Human tau protein (catalog number: orb392120) was purchased from Biorbyt (Cambridge, UK). The DNA aptamers were synthesized and purified by Sangon Biotechnology Co., Ltd. (Shanghai, China). The thiolated aptamers to the AβO and tau protein possessed the sequences of 5′-HS-(CH_2_)_6_-GCC TGT GTT GGG GCG GGT GCG and 5′-HS-(CH_2_)_6_-GCG GAG CGT GGC AGG-3′, respectively. A human serum sample was provided by the Sun Yat-sen University Cancer Center. Ultrapure water (18.2 MΩ cm,) was used to prepare the aqueous solutions throughout the experiments.

### 2.2. Instruments and Characterization

Transmission electron microscopic (TEM) images were taken using a Hitachi 7700 transmission electron microscope (Tokyo, Japan) with an accelerating voltage of 120 kV. The steady-state and transient photoluminescence spectra were measured using an Edinburgh Instruments FS5 spectrometer (Livingston, UK) with the excitation wavelengths at 405 nm and 450 nm, respectively. The absolute photoluminescence quantum yield of the QDs was measured using a QE65000 spectrometer equipped with an Ocean Optics FOIS-1 integrating sphere. The QD sample was gradiently diluted with an optical density below 0.1. Each diluted sample was measured at room temperature. The accuracy and stability of the measurement system were verified using organic dyes with known photoluminescence quantum yields. The ultraviolet-visible (UV-vis) spectra were monitored using an AgilentCary 60 spectrophotometer (Palo Alto, CA, USA). The zeta potential and hydrated particle size were measured at a neutral pH with a Malvern Zen 3600 Zetasizer (Malvern, UK). All the optical measurements were performed at room temperature.

### 2.3. Preparation of Water-Soluble Dual-Color CdSe/CdS/ZnS Core/Shell/Shell QDs

The alkanoate-coated CdSe/CdS/ZnS core/shell/shell QDs were synthesized according to the previous method [44]. The purification process of the core/shell/shell QDs can be described as follows. A total of 1 mL of the reaction mixture was mixed with 5 mL of ethyl acetate, and this was followed by centrifugation at 10,000 rpm for 3 min. The supernatant was removed, and the above procedure was repeated three times. After that, water-soluble mercaptopropionic acid (MPA)-capped CdSe/CdS/ZnS QDs were synthesized by exchanging the carboxylate ligands for MPA, following the reported protocol [52]. Briefly, 50 μL of MPA was added to 2 mL of purified QDs in chloroform. After ultrasonication for 10 min and centrifugation at 4000 rpm for 2 min, the precipitates were washed twice with hexane. Then, the precipitates were dried using high-purity argon to completely remove the organic solvent. Finally, the precipitates were dissolved in 610 μL of aqueous solution containing 10 μL of TMAH, and the concentration of the QDs was estimated to be 20 μM according to Lambert–Beer’s law, in which the extinction coefficient is determined by Peng’s group [53].

### 2.4. Fabrication of the Aptamer-Functionalized QDs

The aptamers to the AβO and tau protein were conjugated with the QDs via a ligand exchange process [41]. Briefly, 200 μL of 10 μM of the aptamer to AβO was mixed with 10 μL of tris (2-carboxyethyl) phosphine hydrochloride (TCEP, 20 mM) at room temperature for 1 h to break the S−S bond between the thiolated aptamers, followed by incubation with 10 μL of the MPA-capped QDs_625_ (20 μM) that emit at 625 nm. The mixed solution was shaken overnight at room temperature under dark conditions, which allowed for the complete exchange of the thiol groups on the MPA-functionalized QDs with the thiolated aptamers. The resultant samples were purified by ultrafiltration using a centrifugal filter with a 50 kDa molecular weight cutoff (centrifugation at 8000 rpm for 2 min for three times). Finally, the upper phase containing the aptamer-capped QDs was redispersed in 2 mL of deionized water and stored at 4 °C for further use. The functionalization of the QDs_665_ that emit at 665 nm with the aptamer to the tau protein was performed in a similar way to that of the QDs_625_.

### 2.5. Synthesis of Au NRs-CTAB

First, the CTAB-capped Au seeds were prepared according to the previous method [54]. Briefly, 7.5 mL of 0.1 M CTAB was mixed with 100 μL of 24 mM HAuCl_4_, and the above solution was diluted with 9.4 mL of deionized water. Then, 0.6 mL of 10 mM ice-cold NaBH_4_ was added under magnetic stirring. After vigorous stirring for 2 min, the seed solution was kept at room temperature and used within 3 h. Afterwards, the growth of the Au NRs was initiated by the introduction of the growth solution (100 mL of 0.1 M CTAB, 2 mL of 24 mM HAuCl_4_, 2 mL of 0.5 M H_2_SO_4_, 350 μL of 10 mM AgNO_3_, and 800 μL of 0.1 M ascorbic acid) into 240 μL of the seed solution under vigorous stirring. The above mixed solution was stirred for 2 min at 30 °C and then left to stand for 12 h. The precipitates were collected by centrifugation at 8000 rpm for 10 min and washed with deionized water more than three times to remove the surfactant and unreacted materials. The obtained Au NRs-CTAB was redispersed in 20 mL of deionized water with a concentration of 1 mg/mL.

### 2.6. Synthesis of Au NRs@PDA

The CTAB-capped Au NRs were unstable in alkaline conditions during dopamine self-polymerization. To solve this problem, a ligand exchange reaction, in which the CTAB layer on the Au NRs was replaced with SH-PEG-CH_3_, was performed [50]. Briefly, 10 mL of the Au NRs-CTAB (1 mg/mL) was added into 1 mL of SH-PEG-CH_3_ (5 mg/mL), followed by sonication in an ice bath for 2 h. By removing the supernatant through two centrifuging procedures at 8000 rpm for 10 min, the precipitates of the Au NRs-PEG were collected and dispersed in 10 mL of deionized water. Then, 2 mL of the obtained Au NRs-PEG was redispersed in 17 mL of tris-HCl solution (5 mM, pH 8.5), followed by the addition of 1 mL of 2 mg/mL dopamine and then sonication for 60 min. The Au NRs@PDA with various shell thicknesses were synthesized by simply changing the polymerization time (30, 60, and 90 min). Finally, by centrifuging twice at 8000 rpm for 10 min, the Au NRs@PDA was redispersed in 4 mL of deionized water with a concentration 0.5 mg/mL.

### 2.7. Formation of Soluble AβO

The treatment of the Aβ40 powder was carried out based on previous work [5]. Briefly, lyophilized Aβ peptide was dissolved in 1 mg/mL of HFIP and incubated overnight at room temperature. The above solution was sonicated for 30 min, and HFIP was evaporated under a gentle nitrogen stream. Then, the Aβ peptide was redissolved in dimethyl sulfoxide (DMSO) at a concentration of 1 mM and stored at −20 °C for later use. The AβO and Aβ fibrils (AβF) were obtained by incubating 100 μM of the pretreated Aβ in phosphate-buffered solution (20 mM, pH 7.4) in a thermostatic water bath at 37 °C for 24 h and 72 h in the dark, respectively. Based on the UV-vis spectra, the concentrations of AβO and AβF were determined and calculated as equivalent to the concentration of the pretreated Aβ peptide.

### 2.8. Simultaneous Detection of AβO and Tau Protein Using the FRET Aptasensor

To detect the AβO and tau protein simultaneously, 10 μL of the AβO-aptamer-functionalized QDs_625_ were mixed with 10 μL of the tau-protein-aptamer-functionalized QDs_665_, and then 55 μL of 0.5 mg/mL Au NRs@PDA was introduced into the mixture to quench the fluorescence upon 30 min of incubation. In the presence of different concentrations of AβO (0, 100, 250, 500, 1000, 2000, 5000, and 10,000 pM) and tau protein (0, 50, 250, 750, 1000, 1500, 3000, and 5000 pM), the recovered fluorescence was recorded upon 120 min of incubation at room temperature. The fluorescence measurement conditions were as follows: excitation wavelength = 405 nm, excitation and emission slit width = 5.0 nm.

## 3. Results

### 3.1. Characterization of the QDs, QDs-DNA Conjugates, and Au NRs

**Figure 1:**
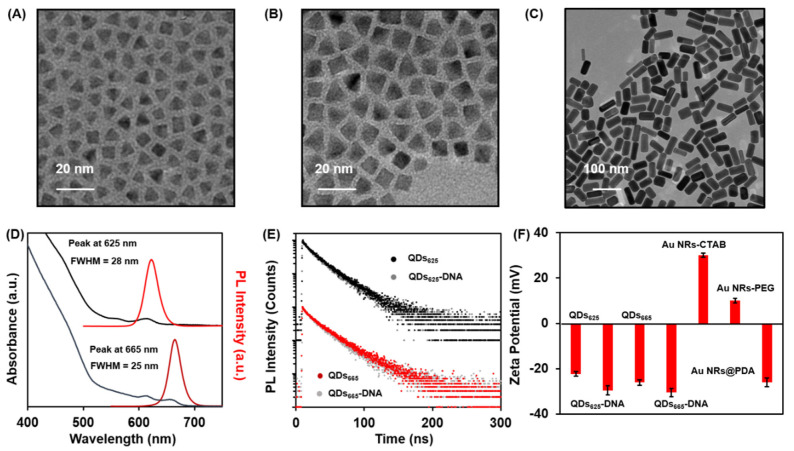
TEM images of (**A**) the alkanoate-coated QDs_625_, (**B**) QDs_665_ in toluene, and (**C**) Au NRs-CTAB. (**D**) UV-vis absorption and fluorescence spectra of the QDs_625_-DNA and QDs_665_-DNA conjugates. The FWHM represented the full width at half maximum. (**E**) PL decay dynamics of QDs_625_ and QDs_665_ before and after conjugation with aptamers that were excited at 450 nm. (**F**) Zeta potentials of QDs_625_, QDs_625_-DNA, QDs_665_, QDs_665_-DNA, Au NRs-CTAB, Au NRs-PEG, and Au NRs@PDA.

Figure 1A–C shows the transmission electron microscopy (TEM) images of the two types of QDs and the corresponding Au NRs. Using CdSe QDs as the core with average diameters of 3.0 nm and 5.4 nm, the QDs_625_ and QDs_665_ were synthesized according to our previous report [44]. The alkanoate-coated CdSe/CdS/ZnS core/shell/shell QDs emitted at 625 nm (QDs_625_) and 665 nm (QDs_665_) and were excited at 405 nm, respectively. The QDs_625_ and QDs_665_ both possessed ideal optical properties toluene (Figure S1A–D) and showed well-controlled interfacial structures, with a small size distribution (Figure 1A,B and Figure S2A,B). As evidenced by the EDS mapping image of Se, the CdSe was located in the center of the core/shell/shell structure, while Zn and S elements were distributed in the outer shell, indicating the well-controlled epitaxial growth of the shell (Figure S3). Moreover, the single crystalline structure of the QDs was observed (Figure S4). The hydrophilic ligand of MPA was used to convert the QDs into water-soluble forms [52,55]. After ligand exchange, the photoluminescence (PL) spectra were still narrow and symmetric, and the emission wavelengths and peak widths remained the same (Figure 1D). The QDs_625_ and QDs_665_ showed a near-unity PL quantum yield (>90%), and the transient PL spectra could be well-fitted by a mono-exponential function (Figure 1E, 1000 counts, goodness-of-fit < 1.30), indicating the elimination of the trap states accessible to the excited states. The CdSe/CdS/ZnS core/shell/shell QDs exhibited a high optical quality in both toluene and water media, which resulted from the additional layer of the inorganic ZnS shell with a large bandgap. The outermost shell could isolate the electron and hole wavefunctions from the environment, thus substantially improving the photostability of the QDs. The excellent optical stability of the water-soluble QDs (Figure S5A,B) provided the basis for their biological applications.

Aptamers can be attached to QDs via ligand exchange between the thiol groups of MPA-functionalized QDs and the thiolated aptamers [56]. Notably, the PL decay dynamics and fluorescence intensities of the QDs_625_ and QDs_665_ were almost unchanged before and after conjugating them with thiolated aptamers (Figure 1E and Figure S6A,B), indicating no additional surface defect states of the QDs upon their association with the aptamers and the preserved ideal optical quality of the QDs-DNA conjugates. Moreover, the successful conjugation of the aptamers to the QDs was confirmed by zeta potential measurements (Figure 1F). The QDs-DNA conjugates possessed a more negative zeta potential than the pure QDs due to the negatively charged phosphate backbone of the aptamers. Furthermore, the hydrodynamic diameter of the QDs_625_ or QDs_665_ functionalized with aptamers was slightly larger than that of the pure QDs (Figure S7A,B), further demonstrating the conjugation of the QDs with the aptamers.

The Au NRs@PDA served as an excellent fluorescence quencher of the QDs [47]. The CTAB-capped Au NRs were prepared by a modified silver-assisted seed growth method, and a typical uniform rod structure with an average diameter of 54 nm was attained (Figure 1C and Figure S8). The Au NRs possessed wide absorption bands centered at 524 and 654 nm (Figure S9), which corresponded to the transverse and longitudinal surface plasmon resonance (SPR) of the Au NRs, respectively [46]. After the replacement of the CTAB with polyethylene glycol (PEG), the surface charge changed from +30.1 to +10.0 mV (Figure 1F), and a slight blue shift of the longitudinal SPR peak was obtained (Figure S9). After coating the PEG-functionalized Au NRs with PDA, the zeta potential changed to −26.2 mV (Figure 1F), which was attributed to the abundant phenolic hydroxyl groups on the PDA shell.

### 3.2. Feasibility of the Proposed Aptasensor

**Figure 2:**
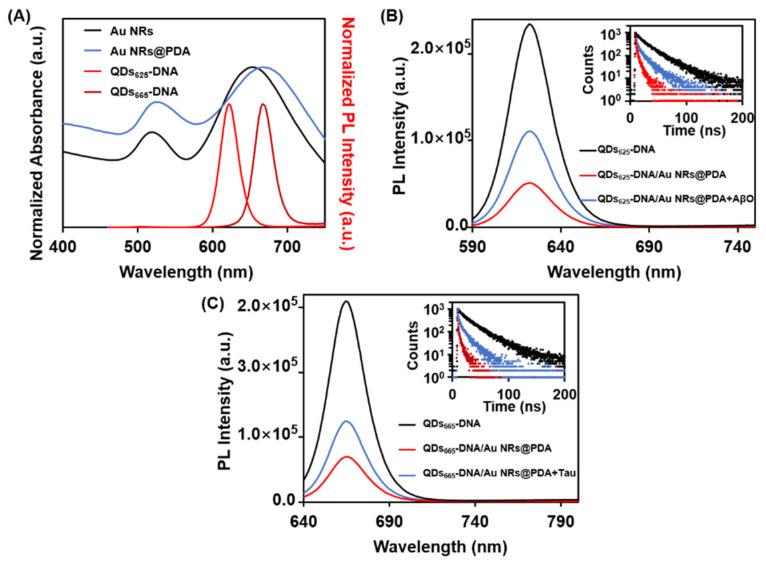
(**A**) The absorption spectra of Au NRs and Au NRs@PDA, and fluorescence spectra of QDs_625_-DNA and QDs_665_-DNA. Feasibility of the aptasensor for the (**B**) AβO and (**C**) tau protein. The insets in B and C show the corresponding PL decay dynamics excited at 450 nm. The concentrations of the AβO and tau protein were maintained at 5 nM and 2 nM, respectively.

As shown in Figure 2A, coating the surface of the Au NRs-PEG with PDA led to a redshift of the longitudinal SPR peak from 645 nm (black curve) to 668 nm (blue curve). In the meantime, the absorption spectrum of the Au NRs@PDA overlapped with the fluorescence spectrum of the QDs_625_-DNA or QDs_665_-DNA, providing the possibility of energy transfer from the QDs to the Au NRs@PDA. As expected, the QDs_625_-DNA showed strong fluorescence upon excitation at 405 nm (black curve, Figure 2B). However, with the incorporation of the Au NRs@PDA, the fluorescence intensity of the QDs_625_-DNA decreased by about 82% (red curve, Figure 2B). Simultaneously, the PL decay dynamics changed from a mono-exponential function (black curve, inset of Figure 2B) to double-exponential function (red curve, inset of Figure 2B). The additional short lifetime decay channel indicated the existence of the efficient nonradiative recombination of the excited states upon binding to the Au NRs@PDA, which led to the reduced PL quantum yield. The fluorescence quenching was induced by the distance-dependent FRET resulting from the binding of the QDs-DNA conjugates (donors) with the Au NRs@PDA nanoparticles (acceptors) via π–π stacking and hydrogen bonding. Due to the presence of amino, hydroxyl, and quinone groups on the PDA, the single-stranded DNA could be attached to the PDA surface. Upon the incubation of AβO with the QDs_625_-DNA/Au NRs@PDA composites, the fluorescence of the QDs_625_ was partially recovered (blue curve, Figure 2B). The specific binding between the AβO and its aptamer thus weakened the interaction between the QDs_625_-DNA and Au NRs@PDA, and the detachment of the QDs_625_-DNA from the surface of the Au NRs@PDA led to the recovery of the fluorescence signal. In the presence of AβO, the fraction of the short lifetime decay channel decreased significantly (blue curve, inset of Figure 2B) relative to that of the QDs_625_-DNA/Au NRs@PDA composites, further demonstrating that the FRET between the QDs_625_-DNA and Au NRs@PDA was destroyed to a large degree. In the case of the tau protein, similar results were attained for the QDs_665_-DNA/Au NRs@PDA composites (Figure 2C). Therefore, the sensing protocol served as a reliable means of the simultaneous and accurate determination of the AβO and tau protein.

### 3.3. Optimization of the Experimental Conditions

**Figure 3:**
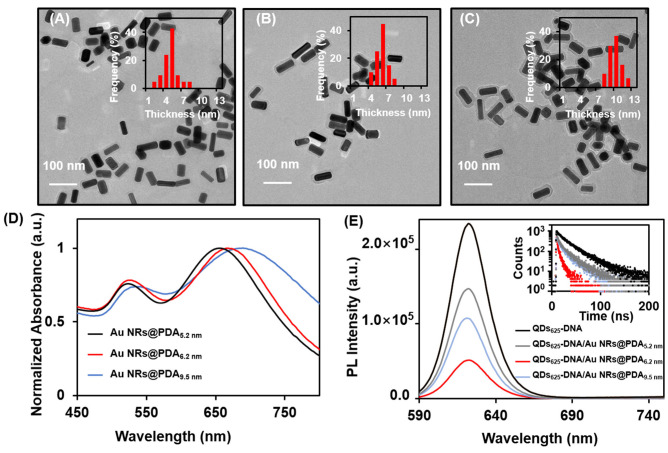
(**A**–**C**) TEM images of Au NRs@PDA with various shell thicknesses. The inset histograms show the average thickness of the PDA shell. (**D**) The absorption spectra of Au NRs@PDA with various shell thicknesses. (**E**) Fluorescence spectra of QDs_625_-DNA in the absence and presence of Au NRs@PDA with various shell thicknesses. The inset shows the corresponding PL decay dynamics.

To achieve a better analytical performance, the self-polymerization time of the dopamine, concentration of the Au NRs@PDA, and incubation time for fluorescence quenching and recovery were optimized. The FRET efficiency was strongly dependent on the distance between donor–acceptor pairs and spectral overlap between the donor emission and acceptor absorption [57]. Notably, the absorption spectrum of the Au NRs@PDA and thickness of the PDA shell could be tuned by adjusting the self-polymerization time of the dopamine. In the weak alkaline medium, the Au NRs@PDA with a uniform and thin PDA shell was constructed via the in situ polymerization of dopamine for different time periods (30, 60, and 90 min for Figure 3A–C, respectively), and the inset histograms in Figure 3A–C show that the average PDA shell thicknesses were 5.2, 6.2, and 9.5 nm, respectively. The Au NRs@PDA with various shell thickness possessed the maximum absorption peaks at 650, 668, and 685 nm, respectively (Figure 3D). Next, the fluorescence quenching ability of the Au NRs@PDA with various shell thicknesses for the QDs_625_-DNA was examined (Figure 3E). Remarkably, a larger fluorescence quenching efficiency was attained when the shell thickness of the PDA was 6.2 nm (red curve), which was accompanied by the significantly changed PL decay dynamics of the QDs_625_-DNA (the inset red curve). However, in the case of the PDA_5.2 nm_, with a thin thickness, the PDA coating might not have been uniform, while the increased thickness of the PDA_9.5 nm_ might have led to the weakened FRET effect. Likewise, similar results were obtained for the QDs_665_-DNA when the shell thickness of the PDA was maintained at 6.2 nm (Figure S10).

The concentration of the Au NRs@PDA was crucial for quenching the fluorescence of the QDs-DNA. As shown in Figure S11A, the fluorescence intensity of the QDs_625_-DNA gradually decreased with the increase in the Au NRs@PDA concentration and began to level off after 25 μg/mL. Thus, 25 μg/mL of the Au NRs@PDA was used in the following experiments. Similarly, the fluorescence intensity of the QDs_665_-DNA decreased with the increasing concentration of the Au NRs@PDA and plateaued at 20.8 μg/mL (Figure S11B). The incubation time also served as an important parameter for the fluorescence quenching and recovery. The fluorescence quenching of the QDs_625_-DNA (Figure S12A) or QDs_665_-DNA (Figure S12B) by the Au NRs@PDA became saturated at 30 min. With the incorporation of the AβO (Figure S12C) or tau protein (Figure S12D), the fluorescence recovery reached a maximum level at 120 min. Thus, the optimized incubation times for the fluorescence quenching and recovery were fixed at 30 min and 120 min, respectively.

### 3.4. Cross-Reactivity Analysis and Simultaneous Detection of the AβO and Tau Protein

**Figure 4:**
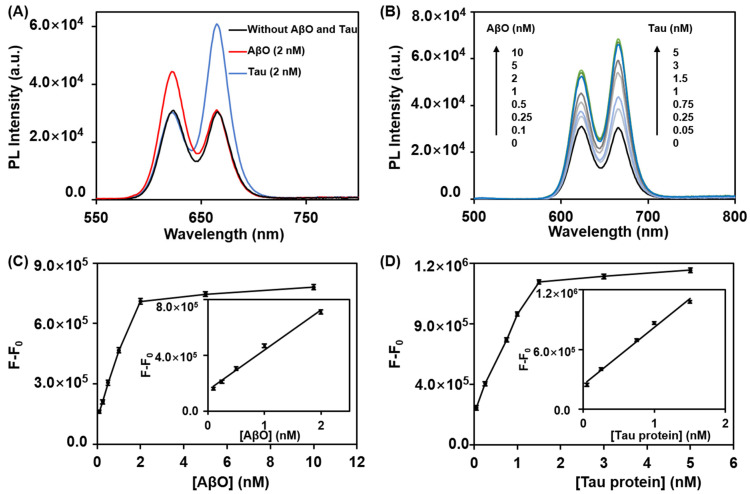
(**A**) Fluorescence spectra of QDs-DNA/Au NRs@PDA composites in the absence and presence of AβO or tau protein. (**B**) Fluorescence spectra of QDs-DNA/Au NRs@PDA composites upon incubation with different concentrations of AβO and tau protein. (**C**) Dependence of the F−F_0_ for QDs_625_-DNA on the concentrations of AβO. The inset shows the linear relationship with the concentrations of AβO from 100 to 2000 pM. (**D**) Dependence of the F−F_0_ for QDs_665_-DNA on the concentrations of tau protein. The inset shows the linear relationship with the concentrations of the tau protein from 50 to 1500 pM.

The CdSe/CdS/ZnS QDs-based FRET aptasensor was capable of simultaneously determining the AβO and tau protein. First, the cross-reactivity between the AβO and tau protein was investigated. As shown in Figure 4A, the fluorescence intensity of theQDs_625_-DNA increased upon the incorporation of AβO, while that of the QDs_665_-DNA remained unchanged. Similarly, the existence of the tau protein only influenced the fluorescence intensity of the QDs_665_-DNA. These results suggested no cross-reactivity between the AβO and tau protein. Under the optimum experimental conditions, the fluorescence intensities of the QDs_625_-DNA and QDs_665_-DNA excited at 405 nm gradually increased with the increasing concentrations of the AβO and tau protein, respectively (Figure 4B). The increased fluorescence intensity could be ascribed to the formation of a protein-aptamer complex, thus increasing the distance between the QDs-DNA and Au NRs@PDA and prohibiting the energy transfer from the QDs-DNA to Au NRs@PDA. The F−F_0_ (where F and F_0_ represent the integral fluorescence area of the QDs_625_-DNA in the presence and absence of AβO, respectively) was found to be linearly proportional to the concentrations of AβO, ranging from 100 to 2000 pM (Figure 4C and the inset), and the linear regression equation was expressed as F−F_0_ = 287,972 C_AβO_ (nM) + 149,804 (R^2^ = 0.9914). The limit of detection (LOD) for AβO was estimated to be 50 pM based on the signal-to-noise ratio of 3. Likewise, a linear relationship between the F-F_0_ (where F and F_0_ represent the integral fluorescence area of the QDs_665_-DNA in the presence and absence of the tau protein, respectively) was observed, and a concentration of the tau protein ranging from 50 to 1500 pM was attained with the linear regression equation of F−F_0_ = 576,773 C_tau_ (nM) + 248,485 (R^2^ = 0.9904) (Figure 4D and the inset). The LOD for the tau protein was calculated to be 20 pM. The relative standard deviation (RSD) was less than 5% for all the measurements, indicating the high precision and good repeatability of the proposed method. The CdSe/CdS/ZnS QDs-based FRET aptasensor for the assay of dual AD biomarkers possessed an excellent analytical performance, being comparable with or superior to the previously reported work (Table S1). Such a good performance could result from the outstanding fluorescence property of QDs and the high fluorescence quenching efficiency of Au NRs@PDA.

### 3.5. Selectivity of the FRET Aptasensor

**Figure 5:**
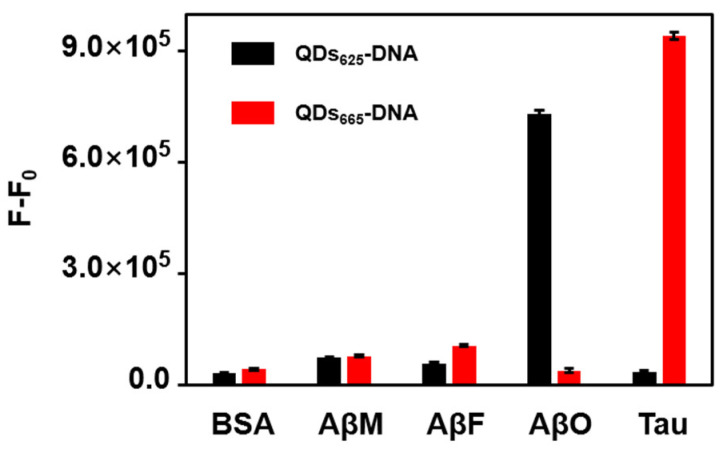
Selectivity of the FRET aptasensor.

To evaluate the specificity of the FRET aptasensor, the interfering species, such as BSA, AβM, and AβF, were investigated. The concentrations of the AβO and tau protein were maintained at 1.5 nM and 1 nM, respectively, while that of the interfering substances was 20 nM. As shown in Figure 5 and Figure S13, although the concentration of the interfering substances was much higher than that of the AβO and tau protein, no obvious fluorescence recovery of the QDs_625_-DNA and QDs_665_-DNA for the interfering species was obtained. The excellent specificity of the proposed method could be ascribed to the high binding affinity between the aptamer and AβO or tau protein.

### 3.6. Real Sample Analysis

To demonstrate the feasibility of the method for potential clinical applications, recovery assays for the AβO and tau protein were carried out. Various concentrations of AβO (200, 500, and 1500 pM) or tau protein (100, 500, and 1000 pM) were added to the 500-fold-diluted human serum samples, and the recovery values of 95–102% for AβO and 98–104% for tau protein were obtained (Table 1), indicating that the method was largely free from the matrix effect of the real sample. The CdSe/CdS/ZnS QDs-based FRET aptasensor thus holds great promise for the multiplexed detection of AD biomarkers in clinical samples.

## 4. Conclusions

The simultaneous detection of dual types of AD core biomarkers was proposed based on a high-quality CdSe/CdS/ZnS QDs FRET aptasensor. Under the optimal conditions, the sensing strategy displayed an excellent analytical performance for the detection of the AβO and tau protein, and the detection limits of 50 pM and 20 pM were obtained, respectively. Compared with the previously reported fluorescent aptasensors, the proposed method possesses the following unique features. Firstly, the distinguishable and robust fluorescence signals of QDs and the broad spectral absorption of Au NRs@PDA provide the basis for the simultaneous detection of two types of AD core biomarkers. Secondly, the sensing protocol is simple and does not involve difficult separation procedures. Furthermore, the FRET aptasensor possesses a high flexibility for the assay of other target analytes using aptamers with different sequences. Given the advantages of its facile manipulation and high sensitivity and specificity, the aptasensor holds great promise for the point-of-care testing of core biomarkers related to AD and cancers.

## Figures and Tables

**Table 1:** Determination of AβO and tau protein in human serum samples (*n* = 3).

Analytes	Spiked (pM)	Found (pM)	Recovery (%)	RSD (%)
	200	190	95	3.59
AβO	500	510	102	4.16
	1500	1470	98	2.35
	100	98	98	4.23
tau protein	500	520	104	3.62
	1000	1010	101	4.76

## Data Availability

The data presented in this study are available on request from the corresponding author.

## Supplementary Materials

The following supporting information can be downloaded at: https://www.mdpi.com/article/10.3390/nano12224031/s1, Figure S1. Normalized fluorescence spectra and PL decay dynamics of QDs_625_ and QDs_665_ in toluene; Figure S2. Size distribution diagram of QDs_625_ and QDs_665_; Figure S3. The EDS elemental mapping images of CdSe/CdS/ZnS core/shell/shell QDs; Figure S4. The high-resolution TEM image of CdSe/CdS/ZnS QDs; Figure S5. Normalized fluorescence intensity of the QDs (A) with the prolongation of the storage time in ambient condition and (B) at different temperatures; Figure S6. Normalized fluorescence spectra of QDs_625_ and QDs_665_ before and after conjugation with thiolated aptamers; Figure S7. The hydrodynamic diameters of QDs_625_ and QDs_665_ before and after conjugation with thiolated aptamers; Figure S8. Size distribution diagram of Au NRs; Figure S9. UV-vis absorption spectra of Au NRs and Au NRs-PEG; Figure S10. Fluorescence spectra of QDs_665_-DNA in the absence and presence of Au NRs@PDA with various shell thicknesses and the corresponding PL decay dynamics; Figure S11. Fluorescence spectra of QDs_625_-DNA and QDs_665_-DNA in the presence of Au NRs@PDA with various concentrations; Figure S12. Influence of the incubation time on fluorescence quenching and recovery; Figure S13. Fluorescence spectra of the FRET aptasensor for assaying the targets and the related interfering species; Table S1. Comparison of the analytical performance of the proposed aptasensor with that of other fluorescent biosensors. References [58,59,60,61] are cited in the Supplementary Materials.
