# Supplementary material for: A High-Quality CdSe/CdS/ZnS Quantum-Dot-Based FRET Aptasensor for the Simultaneous Detection of Two Different Alzheimer’s Disease Core Biomarkers
Source: Nanomaterials (Basel). 2022 Nov 16;12(22):4031. doi: 10.3390/nano12224031 (PMC9697525; doi:10.3390/nano12224031)
Supplement: Supplementary file 1 [file nanomaterials-12-04031-s001.zip › nanomaterials-1967841-supplementary.pdf]

nanomaterials

Supplementary Material

A High-Quality CdSe/CdS/ZnS Quantum-Dot-Based FRET Aptasensor for the Simultaneous Detection of Two Different Alzheimer's Disease Core Biomarkers

Xingchang Lu [1], Xiaoqi Hou [2,3,*], Hailin Tang [4], Xinyao Yi [1] and Jianxiu Wang [1,*]

1 Hunan Provincial Key Laboratory of Micro & Nano Materials Interface Science, College of Chemistry and Chemical Engineering, Central South University, Changsha 410083, China
2 School of Chemistry and Material Science, Hangzhou Institute for Advanced Study, University of Chinese Academy of Sciences, 1 Sub-lane Xiangshan, Hangzhou 310024, China
3 Key Laboratory of Intelligent Sensing Materials and Chip Integration Technology of Zhejiang Province, Hangzhou Innovation Institute, Beihang University, Hangzhou 310052, China
4 SunYat-sen University Cancer Center, State Key Laboratory of Oncology in South China, Collaborative Innovation Center for Cancer Medicine, Guangzhou 510060, China
* Correspondence: houxiaoqi@ucas.ac.cn (X.H.); jxiuwang@csu.edu.cn (J.W.)

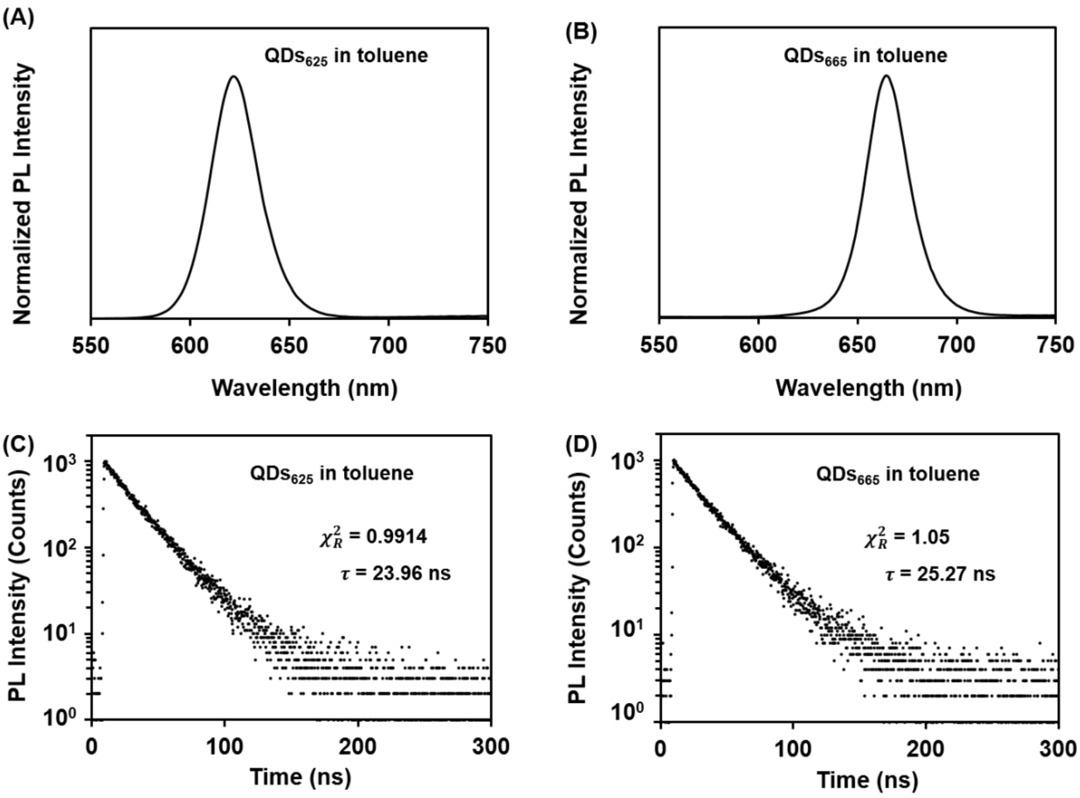

Figure S1. Normalized fluorescence spectra of (**A**) QDs_{625} and (**B**) QDs_{665} in toluene. PL decay dynamics of (**C**) QDs_{625} and (**D**) QDs_{665} in toluene. τ and χ_R^2 represented the mono-exponential fitting lifetime and goodness-of-fit, respectively.

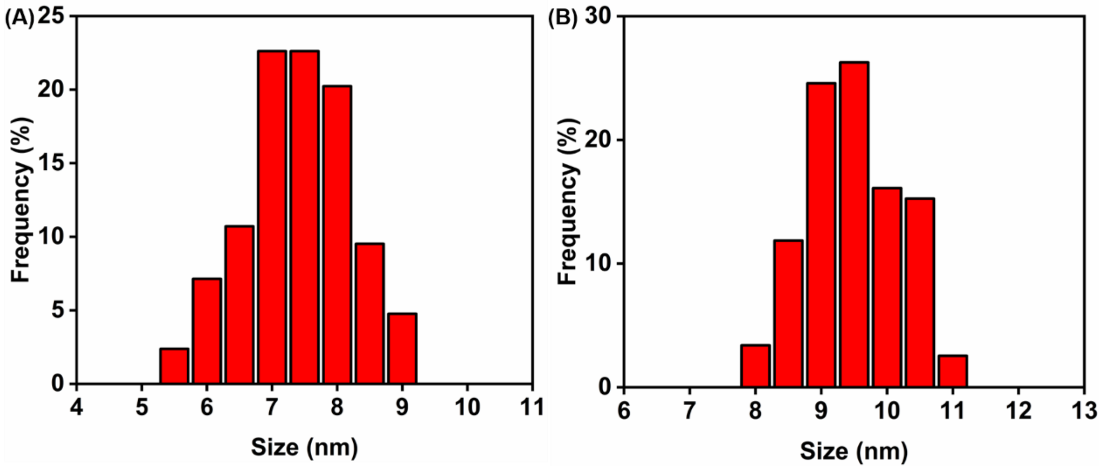

Figure S2. Size-distribution diagram for (**A**) QDs_{625} and (**B**) QDs_{665}.

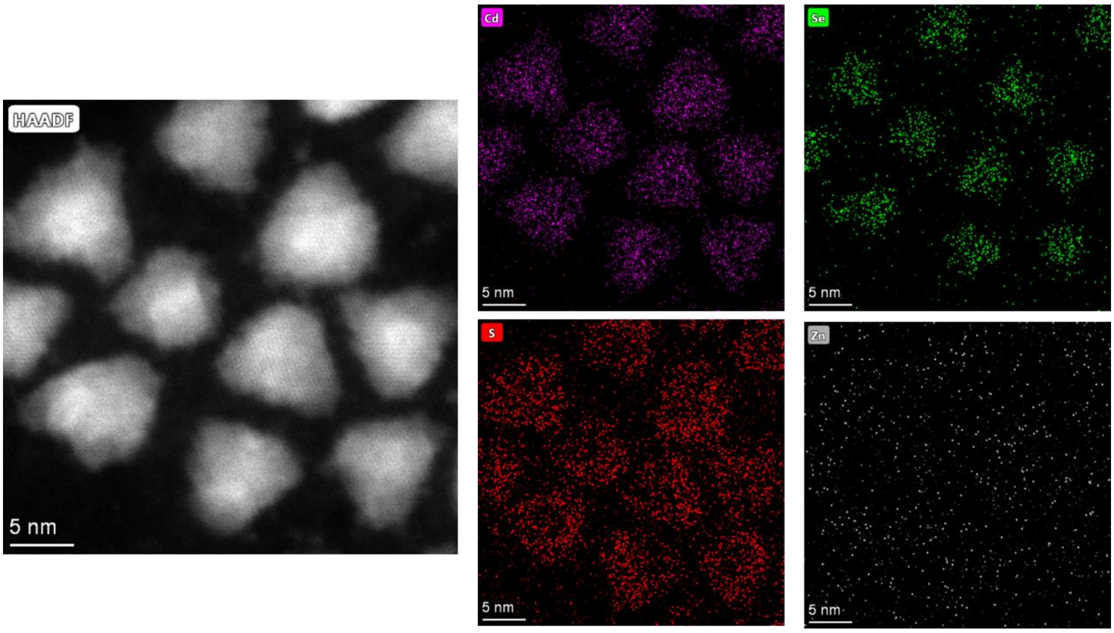

Figure S3. The EDS elemental mapping images of CdSe/CdS/ZnS core/shell/shell QDs.

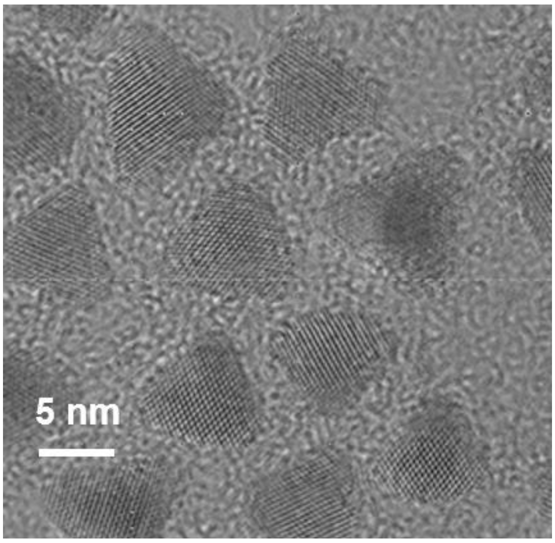

Figure S4. The high-resolution TEM image of CdSe/CdS/ZnS QDs.

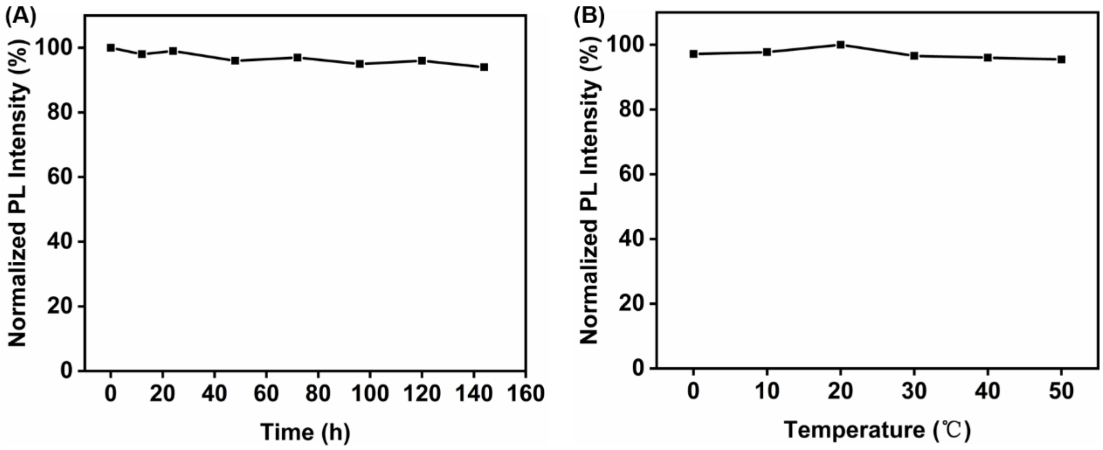

Figure S5. Normalized fluorescence intensity of the QDs (**A**) with the prolongation of storage time in ambient condition and (**B**) at different temperatures.

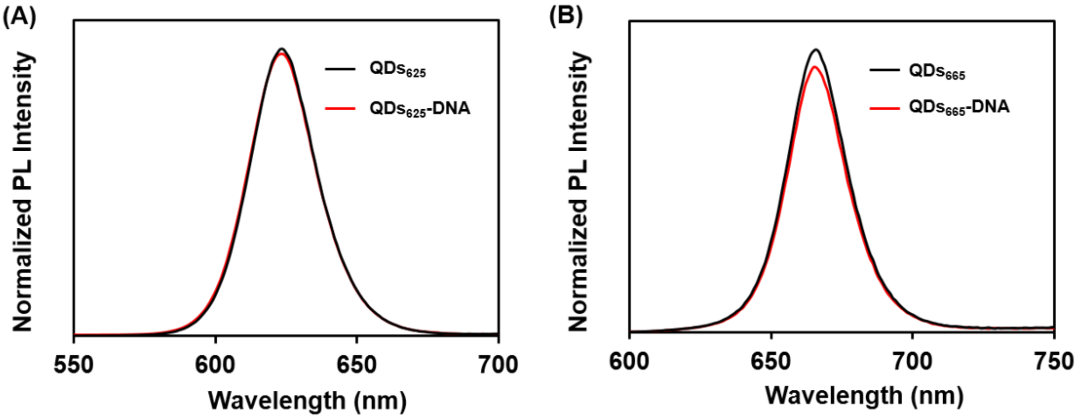

Figure S6. Normalized fluorescence spectra of (**A**) QDs_{625} and (**B**) QDs_{665} before and after conjugation with thiolated aptamers toward AβO and tau protein.

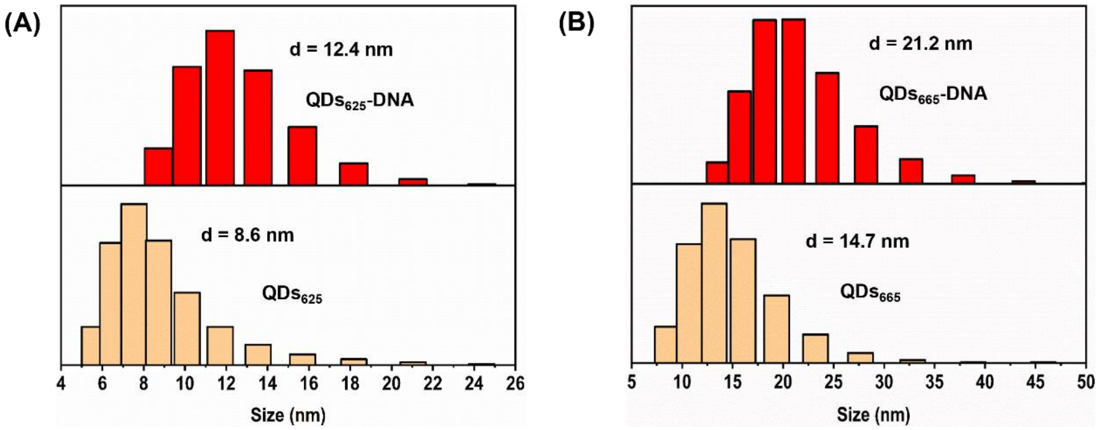

Figure S7. The hydrodynamic diameters of (**A**) QDs_{625} and (**B**) QDs_{665} before and after conjugation with thiolated aptamers.

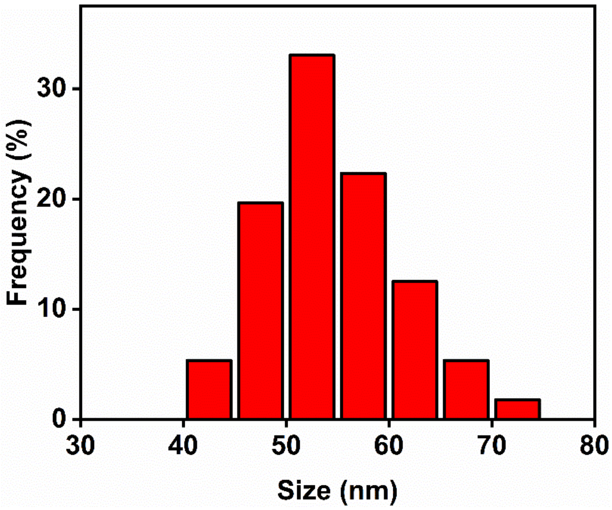

Figure S8. Size-distribution diagram for Au NRs.

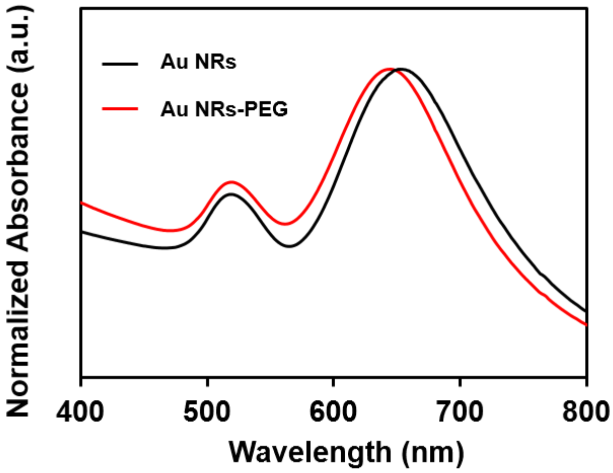

Figure S9. UV-vis absorption spectra of Au NRs and Au NRs-PEG.

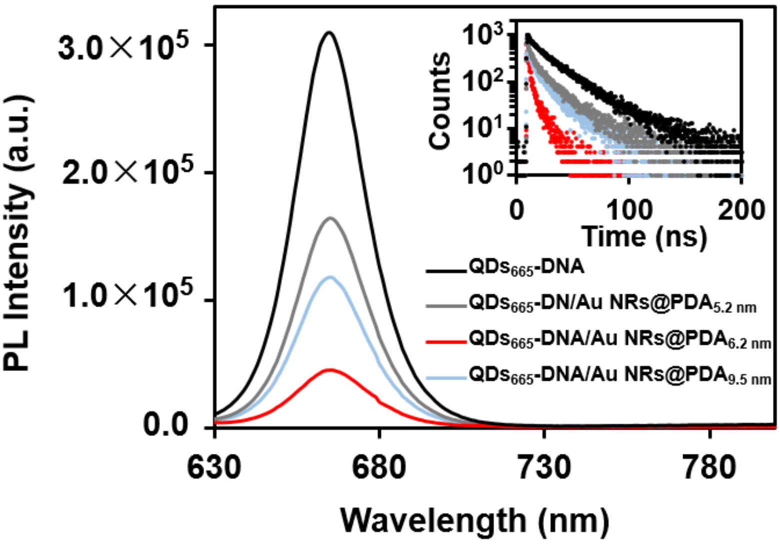

Figure S10. Fluorescence spectra of QDs_{665}-DNA in the absence and presence of Au NRs@PDA with various shell thicknesses. The inset showed the corresponding PL decay dynamics.

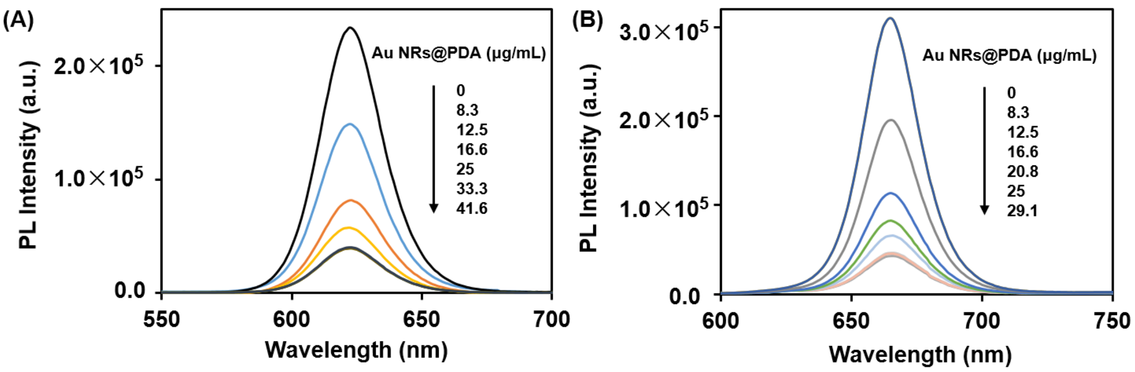

Figure S11. (**A**) Fluorescence spectra of QDs_{625}-DNA in the presence of Au NRs@PDA with various concentrations (0, 8.3, 12.5, 16.6, 25, 33.3 and 41.6 μg/mL). (**B**) Fluorescence spectra of QDs_{665}-DNA in the presence of Au NRs@PDA with various concentrations (0, 8.3, 12.5, 16.6, 20.8, 25 and 29.1 μg/mL).

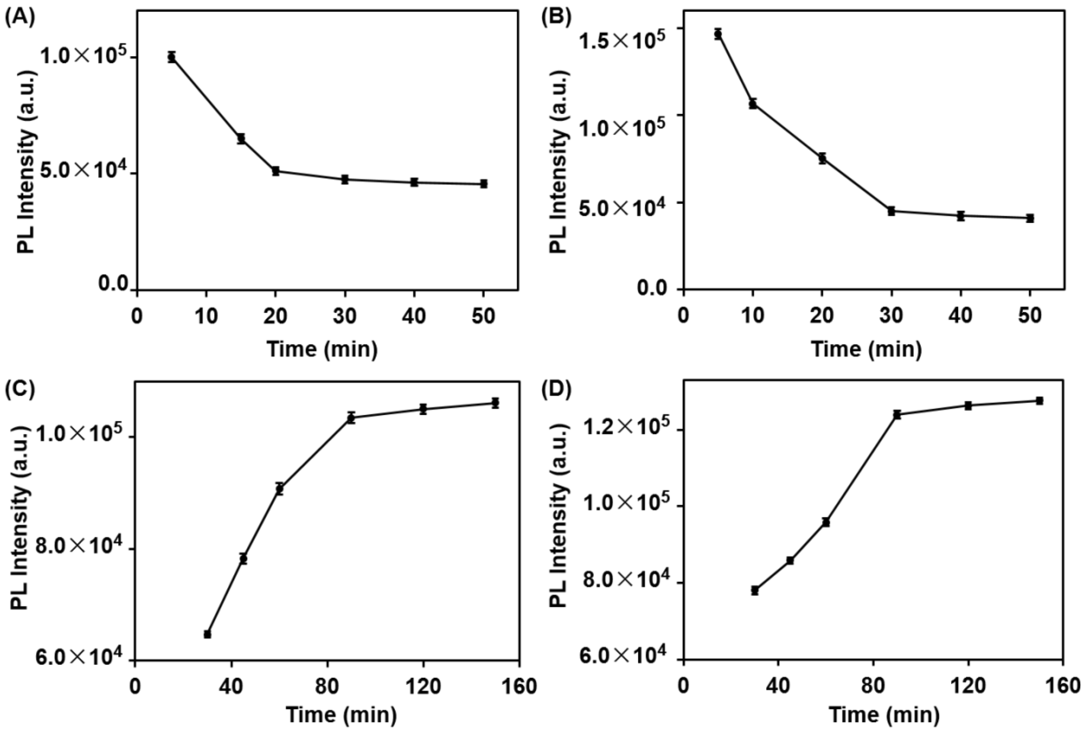

Figure S12. Influence of the incubation time on fluorescence quenching of (**A**) QDs_{625}-DNA and (**B**) QDs_{665}-DNA by Au NRs@PDA. Influence of the incubation time on fluorescence recovery upon incorporation of (**C**) AβO and (**D**) tau protein.

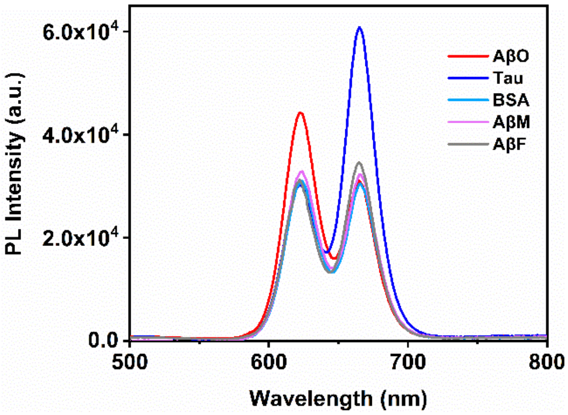

Figure S13. Fluorescence spectra of the FRET aptasensor for assaying the targets and related interfering species.

Table S1. Comparison of the analytical performances of the proposed aptasensor with those of other fluorescent biosensors.

detection mechanism	biomarker	linear range	detection limit	reference
PDANS/FAM-DNA nanocomplexes as the probes	AβO	0.02–10 µM	12.5 nM	[1]
Immobilization of dye-labeled aptamer on the GO surface	AβO	0.1–40 nM	0.1 nM	[2]
Adsorption of FAM-labeled aptamer on the surface of PBNPs	AβO	1–100 nM	1 nM	[3]
Interaction of aptamer-functionalized Fe_3O_4 NPs with UCNPs	AβO	0.2–15 nM	36 pM	[4]
Tyrosinase-induced tau aptamer-tau-tau antibody sandwich immunoassay	tau	0.01–200 nM	9.7 pM	[5]
GO-based competitive immunoassay	tau	2–20 ng/mL	0.14 pM	[6]
Interaction of aptamers-functionalized multicolor QDs with Au NRs@PDA	AβO<br>tau	0.1–2 nM<br>0.05–1.5 nM	50 pM<br>20 pM	This work

References

1. Liu, L.; Chang, Y.; Yu, J.; Jiang, M.; Xia, N. Two-in-one polydopamine nanospheres for fluorescent determination of beta-amyloid oligomers and inhibition of beta-amyloid aggregation. *Sens. Actuators B Chem.* **2017**, *251*, 359-365.
2. Zhao, Y.; Li, X.; Yang, Y.; Si, S.; Deng, C.; Wu, H. A simple aptasensor for Aβ40 oligomers based on tunable mismatched base pairs of dsDNA and graphene oxide. *Biosens. Bioelectron.* **2020**, *149*, 111840.
3. Chen, W.; Gao, G.; Jin, Y.; Deng, C. A facile biosensor for $Aβ_{40}O$ based on fluorescence quenching of prussian blue nanoparticles. *Talanta* **2020**, *216*, 120930.
4. Jiang, L.; Chen, B.; Chen, B.; Li, X.; Liao, H.; Huang, H.; Guo, Z.; Zhang, W.; Wu, L. Detection of Aβ oligomers based on magnetic-field-assisted separation of aptamer-functionalized Fe_3O_4 magnetic nanoparticles and $BaYF_5$:Yb,Er nanoparticles as upconversion fluorescence labels. *Talanta* **2017**, *170*, 350-357.
5. Chen, L.; Lin, J.; Yi, J.; Weng, Q.; Zhou, Y.; Han, Z.; Li, C.; Chen, J.; Zhang, Q. A tyrosinase-induced fluorescence immunoassay for detection of tau protein using dopamine-functionalized $CuInS_2$/ZnS quantum dots. *Anal. Bioanal. Chem.* **2019**, *411*, 5277-5285.
6. Huang, A.; Zhang, L.; Li, W.; Ma, Z.; Shuo, S.; Yao, T. Controlled fluorescence quenching by antibody-conjugated graphene oxide to measure tau protein. *R. Soc. Open Sci.* **2018**, *5*, 171808.
